# Fetus Conceived via In Vitro Fertilization With Mosaic Uniparental Isodisomy and Two Balanced Translocations

**DOI:** 10.7759/cureus.62095

**Published:** 2024-06-10

**Authors:** Ashley E Lall, Samantha Brener, Daniel P Eller

**Affiliations:** 1 Obstetrics and Gynecology, Wellstar Kennestone Hospital, Marietta, USA; 2 Pediatrics and Neonatology, Medical College of Georgia, Augusta University, Augusta, USA; 3 Maternal-Fetal Medicine, Wellstar Kennestone Hospital, Marietta, USA

**Keywords:** mosaicism, case report, imprinting disorders, uniparental isodisomy, balanced translocations

## Abstract

We present a case of a fetus acquiring two different balanced translocations from each parent and subsequent uniparental isodisomy from postzygotic loss of a paternal chromosome. Balanced chromosomal translocations occur in 0.14% of the population and increase the risk of other genetic abnormalities, such as uniparental disomy (UPD) and mosaicism. Preimplantation genetic testing (PGT) can identify some genetic abnormalities. Translocations t(6;21) and t(5;15) have been reported individually but never together in a viable fetus. A non-consanguineous couple who were known carriers of two different balanced translocations conceived via classic in vitro fertilization (IVF). They had a normal PGT completed. Chorionic villus sampling (CVS) revealed that the fetus had received t(6;21) from the mother and t(5;15) from the father. The probability of the fetus acquiring both translocations was 2.8%. CVS also revealed UPD of chromosome 14. Amniocentesis was performed, which was consistent with the CVS in detecting the balanced translocations but provided more information about the UPD, determining that it was a mosaic maternal uniparental isodisomy of chromosome 14 (UPD(14)mat). The couple underwent genetic counseling to discuss the above findings and ultimately decided on dilation and evacuation at 17 weeks of gestation. The likelihood of conception of this fetus and survival past the first trimester is extremely rare. These specific chromosomal translocations and (UPD(14)mat) have never been reported before. This case emphasizes the concomitant nature of imprinted genes, resulting in multiple genetically unique alterations. This report also highlights the limitations of PGT, CVS, and amniocentesis in being reproducibly consistent, which is important to discuss prior to IVF conception.

## Introduction

Balanced chromosomal translocations are present in 0.14% of the population. A balanced chromosomal translocation occurs when two chromosomes exchange segments without gaining or losing genetic material. While carriers of these translocations are often phenotypically normal, reduced fertility is common due to meiotic chromosomal abnormalities [1].

Balanced translocation carriers are at increased risk for uniparental disomy (UPD) and mosaicism in their offspring. UPD is the inheritance of both chromosome homologs from a single parent rather than one homolog from each parent. UPD can be either a heterodisomy, in which the inherited chromosomes are a pair of homologous chromosomes from one parent, or an isodisomy, in which they are identical copies of one parental chromosome. Mosaicism is defined as the co-existence of cells within one organism that contain multiple different chromosomal makeups. This phenomenon occurs from postzygotic mitotic events, most commonly sister chromatid segregation errors [2].

Preimplantation genetic testing (PGT) is useful in reducing pregnancy losses in translocation carriers undergoing in vitro fertilization (IVF). PGT can identify single-gene disorders, translocations, aneuploidies, and mosaicism. In the 1990s, PGT utilized fluorescent in situ hybridization (FISH) to detect chromosomal abnormalities. FISH is still used to identify structural rearrangements, such as translocations, but more advanced techniques involving whole genome amplification and polymerase chain reaction (PCR) have been developed to better identify aneuploidies and other abnormalities. It must be noted that standard PGT cannot differentiate embryos that are carriers of balanced translocations from euploid embryos. A more advanced version of PGT is required for this differentiation. Amniocentesis and chorionic villus sampling (CVS) can further elucidate the results of PGT [3].

The precise translocations presented in this case (t(5;15)(q32;q25) and t(6;21)(p21.3;q22.3)) have never been reported in the literature, neither separately nor together. Translocations of different loci of these chromosomes have been reported, however. Translocation between chromosomes 5 and 15 (t(5;15)) has been associated with acute lymphoblastic leukemia, and translocation between chromosomes 6 and 21 (t;(6;21)) has been associated with acute myeloid leukemia [4,5]. T(6;21) has also been associated with primary infertility, likely due to abnormal spermatocyte meiosis results [6].

The phenotype associated with mosaic maternal isodisomy of chromosome 14q32 presented in this case is called Temple syndrome. Temple syndrome presents with low birth weight, hypotonia, premature puberty, and mild facial dysmorphisms, along with other anomalies. Maternal uniparental isodisomy of chromosome 14q32 (deletions on the paternal chromosome) results in Temple syndrome, whereas paternal uniparental isodisomy (deletions on the maternal chromosome) results in Kagami-Ogata syndrome, which presents with respiratory distress during delivery due to abnormal formation of the thorax, ribs, chest wall, and abdominal wall, among other dysmorphic features [7].

## Case presentation

We present the case of a 35-year-old woman, G4P0121, who conceived via classic IVF. The patient and her partner are known carriers of two different balanced translocations: maternal t(6;21) and paternal t(5;15). The non-consanguineous couple completed standard PGT with their IVF provider, and the results were reportedly normal. An early nuchal translucency was performed, which was also normal. Given that each parent was a carrier of a balanced translocation, a decision was made to forgo additional noninvasive prenatal testing and proceed with CVS and chromosomal microarray testing. This revealed that the female fetus had a translocation between the long arm of one chromosome 5 and the long arm of one chromosome 15, as well as a translocation between the short arm of one chromosome 6 and the long arm of one chromosome 21. Additionally, the unexpected findings of mosaic UPD of chromosome 14 and mosaic duplication on chromosome 3q29 were noted on the CVS. After discussion of the results of the CVS, the couple proceeded with genetic counseling. Through this counseling and discussion with a genetic counselor who reviewed the CVS results, the patient elected to undergo an amniocentesis for more information about the mosaic UPD.

Amniocentesis was performed at the maternal-fetal medicine office later that week without complications. Whole genome sequencing of fetal DNA with Illumina DNA PCR-Free Prep of the amniotic fluid sample revealed mosaic maternal isodisomy of chromosome 14. There was no maternal cell contamination of the results. The report from the amniocentesis discussed that the variant found is a uniparental isodisomy from maternal origin with approximately 70% mosaicism. This finding was noted to occur similarly in patients affected by Temple syndrome. The specific assay used with reported sensitivity, specificity, and positive predictive value (PPV) was also discussed in the final report. The sensitivity, specificity, and PPV for single-nucleotide variants were >0.99. The sensitivity for copy number variants greater than 300 base pairs was >0.96 and >0.99 for short tandem repeats. The amniocentesis was run using the Variantyx Genomic Intelligence database. This system only reports structural variants that are genome-wide and are likely pathogenic with clinical correlation. Parental inheritance was determined via the analysis of parental blood samples along with the amniotic fluid sample. Regions of homozygosity were detected specifically for imprinted chromosomes (6, 7, 11, 14, 15, and 20).

On a follow-up visit, a detailed ultrasound was performed at 16 weeks of gestation, which revealed normal fetal anatomy, but not all fetal anatomic structures were visualized, including the lips, profile, heart, and all extremities. Ultimately, once all of the information was collected, the couple proceeded with dilation and evacuation at 17 weeks of gestation in Maryland.

## Discussion

Imprinting disorders of clinical significance typically occur with alterations of chromosomes 6, 7, 11, 14, 15, 16, and 20. These specific congenital disorders have been known to manifest as a result of UPD events [8]. Our knowledge about UPD has grown rapidly over the past 40 years since the first discussions involving chromosome 15 and Prader-Willi syndrome (PWS) and Angelman syndrome (AS). Both of these disorders occur through UPD of chromosome 15, causing maternal expression and paternal expression in PWS and AS, respectively. The pathophysiology of imprinting involves three crucial steps: erasure, re-establishment during gametogenesis, and maintenance post-fertilization. This process starts with genome-wide demethylation to clean the slate of imprinted loci, followed by parental-specific imprints that are re-established through methylation mechanisms in accordance with the sex of the gamete. With fertilization, the newly formed zygote will have preserved regions of imprints that will continue to be maintained through methylation. Deviations in this closely regulated process allow for imprinting disorders [9].

**Figure 1:**
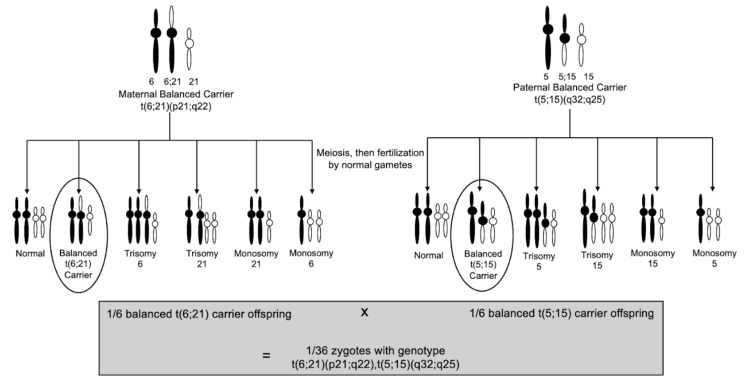
Genetic probability of the fetus acquiring genotype of t(5;15)(q32;q25), t(6;21)(p21.3;q22.3). Image credit: Samantha Brener.

To the best of our knowledge, the translocations in this case have never been reported in the literature. Given that each parent is a balanced carrier for a different translocation, the probability that the fetus acquired both balanced translocations is 1/36 or approximately 2.8% (Figure 1). However, in addition to receiving both balanced translocations from each parent, the acquired maternal UPD of chromosome 14 (UPD(14)mat) makes this case even more novel. There is no exact reported percentage, but the risk of UPD in a fetus who has inherited a translocation of chromosome 14 is rare [10]. However, the maternal karyotype for this case was not significant for any abnormalities with chromosome 14, making this finding even more unlikely. 

The proposed methods for UPD(14)mat include trisomic rescue, monosomic rescue, gamete complementation, and postzygotic errors. Given that a uniparental isodisomy occurred with 70% mosaicism, monosomic rescue and gamete complementation are not possible explanations for how the fetus acquired UPD(14)mat. Monosomic rescue would not result in mosaicism, and gamete complementation would result in heterodisomy. Additionally, with the fetal karyotype not significant for trisomy 14 and the CVS results ruling out placental mosaicism, trisomic rescue is also not a plausible explanation. Thus, it is likely that UPD(14)mat was a result of a postzygotic loss of the paternal chromosome and duplication of the remaining maternal chromosome [11-13].

## Conclusions

This case demonstrates the increased risk of concomitant genetic abnormalities with translocations, such as uniparental isodisomy. It is imperative for patients to understand how their carrier status may impact other genes. The limitations of PGT are evident in this case, given that this fetus with multiple genetic abnormalities had a normal PGT result. Furthermore, even though the CVS had shown mosaic duplication on chromosome 3q29, this result was not found with amniocentesis. The inconsistency among results from PGT, CVS, and amniocentesis emphasizes the need for improved testing methods. Before these methods are fully developed, patients undergoing fertility counseling should be advised that the results of these testing methods are not completely predictive.
